# Use of generic medicines in Latvia: awareness, opinions and experiences of the population

**DOI:** 10.1186/s40545-018-0159-5

**Published:** 2019-01-06

**Authors:** Ieva Salmane Kulikovska, Elita Poplavska, Marija Ceha, Signe Mezinska

**Affiliations:** 1 Faculty of Pharmacy, Riga Stradins University, 16 Dzirciema Str., Riga, LV1007 Latvia; 2 Faculty of Pharmacy and Institute of Public Health, Riga Stradins University, 16 Dzirciema Str., Riga, LV1007 Latvia; 3 Faculty of Pharmacy, Riga Stradins University, 16 Dzirciema Str., Riga, LV1007 Latvia; 4 Faculty of Medicine and Institute of Clinical and Preventive Medicine University of Latvia, 19 Raina Blvd., Riga, LV1586 Latvia

## Abstract

**Background:**

To stimulate use of generic medicines a combination of supply and demand side mechanisms are employed in the Latvian reimbursement system. It is reported that patients have high out-of-pocket pharmaceutical spending and that they overpay by not choosing generic medicines. Patient preferences may be an important obstacle in implementing generic policy. Objective of this study was to assess awareness, opinions and experience of the Latvian population regarding use of generic medicines.

**Methods:**

Survey of representative sample of the population of Latvia (*n* = 1005) aged 18–74 was conducted in March 2015. The survey was distributed in Latvian and Russian languages using Computer Assisted Web Interviews. Associations between experience with generic medicines, preference for medicines, and sociodemographic variables were tested with Pearson Chi-square statistics. Associations between the previous experience and information given by different sources versus choice between medicines were tested with Spearman’s correlation test.

**Results:**

72.3% of the population were informed about generic medicines. Men (66.9%) and respondents with primary or secondary education (58.3%; 69.3%) were less informed compared to total (72.3%). From those who recalled using generic medicines (*n* = 441), 94.4% evaluated their experience as positive or neutral. Despite this, only 21% of the population would opt for generic medicines. The strongest preference for brand-name medicines was in the age group > 55 (40.5%). Opinion of a physician was the most important factor when choosing between generic and brand-name medicines (88.7%). The more positive the information provided by general practitioners, physician specialists, pharmacists, family members, friends and internet is perceived, the more likely respondents are to choose generic medicines (*p* < 0.001).

**Conclusion:**

This study demonstrates that people in Latvia are aware of generic medicines but only a minority of the population would choose them when presented with a choice. It is therefore important that health care professionals provide objective and unbiased information about generic medicines to their patients. Interventions should aim to reach groups that are less informed and to improve providers’ understanding and communication with patients about generics.

## Introduction

Use of generic medicines (GM) is an important tool to stimulate competition in the pharmaceutical market and reduce the cost of medicines [1, 2]. The consumption of GM in Europe differs among countries, and it is estimated to range between 10 and 90% of the total medicines market [2]. Latvia has a relatively high penetration rate of GM (64% by volume and 33% by value) [3]. To promote use of GM, a combination of supply and demand side mechanisms [4, 5] such as reference pricing, international non-proprietary name (INN) prescribing, generic substitution [6] and public information campaigns is employed within the Latvian reimbursement system. Prescription medications are reimbursed as part of a benefit package in a single-purchaser national health system funded by general tax revenues. The National Health Service of the Republic of Latvia (NHSRL), an administrative institution of the Ministry of Health, produces and maintains a positive outpatient reimbursement list [7]. The list contains approximately 1600 medications and accounts for 39% of total pharmaceutical expenditure. According to the severity of illness and dependency on treatment, medications in the list have three levels of reimbursement - 100, 75% or 50% [6–8]. For the medications that are reimbursed less than 100%, patients cover the remaining cost. Medicines in the reimbursement list are classified into three groups – A, B and C. List A is a reference list of interchangeable medications, but lists B and C contain non-interchangeable, mostly patented products [6–8]. Reference pricing, INN prescribing and mandatory generic substitution are used to encourage use of reference products within the list A. The current regulation specifies that unless there is a documented medical need, in the case where the patient or the prescriber opposes generic substitution, the patient must cover the difference in price between the reference product and the chosen product or in some cases even pay the full price of the chosen medicine. Although access to medicines can partly be hampered by the limited number of medicines covered by the reimbursement list and levels of reimbursement, the NHSRL has estimated that within the Latvian reimbursement system, patients in co-payments overpay several millions a year for medicines by not choosing cheapest available GM [9]. Patients’ preferences might be an important obstacle in implementing effective generic policy [10, 11]. Some individual studies have shown that lay people have no objections to using GM [12] and that they perceive no difference between GM and brand-name medicines [13, 14]. However, the authors of two systematic reviews concluded that high proportion of lay people tend to believe that GM are of lower quality and less effective than brand-name medicines as a result they may feel negatively about generic substitution [15, 16]. The analysis of patients’ opinions has suggested that the most common reason for refusing GM was the customers’ positive experiences with medicines they had used previously, as well as their wish to talk with their doctor before accepting substitutes [17] In Latvia out-of-pocket spending for pharmaceuticals is the second highest among Organization for Economic Co-operation and Development (OECD) countries (39%) [18], many people report unmet health needs [19] and financial difficulties to obtain the prescription medicines they need to manage chronic conditions [20]. Given this situtation the study regarding population’s choices and preferences of GM may offer valuable insights to strengthen generic policy and improve access to medicines.

### Aim of the study

The aim of this study was to assess awareness, opinions and experience of the general population regarding use of GM in Latvia.

## Method

The respondents who participated in the study (*n* = 1005) represented the adult general population of Latvia aged 18 to 74. The sample was selected using the database of the research company SKDS and composed to meet the characteristics of a nationally representative sample. To adjust precisely to the criteria of a representative sample, data were weighted according to four parameters – gender, age, nationality and place of residence, according to the data in the electronic system of the Population Register maintained by the Office of Citizenship and Migration Affairs. The survey was distributed in Latvian and Russian languages using Computer Assisted Web Interviews as part of the monthly Web-Omnibus survey. The data collection took place in March 2015. The dataset generated and analysed during the current study is available in the Open Science Framework repository.

With the authors’ permission, we developed the survey based on the questionnaire by Drozdowska and Hermanowski [21]. The questionnaire was translated into Latvian; we performed a cognitive testing of the questionnaire (*n* = 10) – questions were evaluated in terms of quality and comprehension.

In addition to demographic information, the survey contained the following questions: 1) awareness of GM (“Did you know about the existence of GM?”); 2) experience with GM (“What is your experience with GM? - positive/more positive; neutral; negative/more negative; hard to say”); 3) preference for GM vs brand-name medicines (“If presented with choice, which one would you chose – GM or brand-name medicine?”); 4) factors of importance when choosing generic or brand-name medicines (“When choosing medicines, what is the importance of the following factors – price; producer of a medicine; country in which medicine is produced; information in press, TV and radio; information on the Internet; opinion of a physician; opinion of a pharmacist; opinion of family members; opinion of friends and relatives; my own experience?”; 5) information about GM provided by different sources (GP; physicians-specialists; pharmacists; relatives and friends; information in press, information on the Internet – positive/more positive; neutral; negative/more negative; no information).The answers were measured on a Likert scale (for the purpose of the analysis, we combined some answers, e.g. “very important” and “rather important” was combined as “very/rather important” and “positive” and “rather positive” was combined as “positive/rather positive”).

Data analysis was performed in SPSS v.25 for Windows. The study employed descriptive statistics methods. To test associations between the following variables - experience with GM, preference for medicines, as well as sociodemographic variables - Pearson Chi-square statistics with significance level *p* < 0.05 was used. To normalize data in Chi-square hypothesis testing, we used a standardized residuals ratio (difference between the observed count and the expected count and standard deviation of the expected count). The results were interpreted as follows: if the residual is less than − 1.96, the cell’s observed frequency is less than the expected frequency; if the residual is greater than 1.96, the cell’s observed frequency is greater than the expected frequency.

To test associations between the previous experience and information given by different sources versus choice between medicines, we used Spearman’s correlation test.

## Results

### Awareness about generic medicines

A large part of the population in Latvia (72.3%) was informed about GM (Table 1). There were more informed respondents in the female group (77.3%) than in the male group (66.9%) and within an age group > 55 years (76.9%), if compared to the total number of the informed group. 22.5% of the population were not informed about the existence of GM, and more of those who were not informed were found in young people group (age group 18–24 years) – 35.0%.

**Table 1:** Study population characteristics in terms of knowledge and preferences of GM^a^

Sociodemographic characteristics	Are informed about GM	Are not informed about GM	No opinion
From total (*n* = 1005)	727 (72.3%)	226 (22.5%)	52 (5.2%)
Gender			
Female, *n* = 528	**408 (77.3%)**	**96 (18.2%)**	24 (4.5%)
Male, *n* = 477	**319 (66.9%)**	**130 (27.3%)**	28 (5.8%)
Age			
18–24, *n* = 120	**71 (59.2%)**	**42 (35.0%)**	7 (5.8%)
25–34, *n* = 207	155 (74.9%)	47 (22.7%)	5 (2.4%)
35–44, *n* = 188	141 (75.0%)	39 (20.7%)	8 (4.3%)
45–54, *n* = 192	130 (67.7%)	50 (26.0%)	12 (6.3%)
> 55, *n* = 298	**229 (76.9%)**	**49 (16.4%)**	20 (6.7%)
Education			
Primary, *n* = 24	14 (58.3%)	10 (41.7%)	0
Secondary, *n* = 339	235 (69.3%)	85 (25.1%)	19 (5.6%)
Higher, *n* = 642	478 **(74.5%)**	131 **(20.4%)**	33 (5.1%)
Sociodemographic characteristics	Would prefer/rather prefer GM	Would prefer/rather prefer brand-name medicine	No difference/do not have an opinion
From total (*n* = 1005)	211 (21.0%)	287 (28.6%)	507 (50.4%)
Age			
18–24, *n* = 120	24 **(**20.2%)	**24 (19.3%)**	**72 (60.5%)**
25–34, *n* = 207	43 (20.8%)	**47 (22.7%)**	**117 (56.5%)**
35–44, *n* = 188	47 (25.0%)	**41 (21.8%)**	100 (53.2%)
45–54, *n* = 192	**50 (26.0%)**	55 (28.6%)	87 (45.3%)
> 55, *n* = 298	**47 (15.7%)**	**121 (40.5%)**	**130 (43.8%)**
Nationality			
Latvian, *n* = 586	**107 (18.3%)**	174 (29.7%)	305 (52.0%)
Other, *n* = 419	**104 (24.8%)**	113 (27.0%)	202 (50.4%)

^a^Bolded text refers to responses that differ significantly from the total (adjusted standardized residuals value > 1.96 or < − 1.96)

### Experience with generic medicines

From those respondents who have used GM (*n* = 441; 44%), the majority (55.7%) recognized their experience with GM as positive or more positive than negative, and 38.7% rated their experience as neutral (Table 2). Only 5.7% described their experience as negative or rather negative. No statistically significant changes were detected among different gender and age groups.

**Table 2:** Experience with generic medicines, *n*=441^b^

	*n*	%
Negative or more negative than positive	25	5.7
Neutral	170	38.7
Positive or more positive than negative	246	55.7
Total	441	100.0

^b^Respondents who have not used GM were excluded from the analysis

### Preferences of the respondents

As seen in Tables 1, 21.0% of the respondents would rather or definitely choose GM; 28.6% would rather or definitely opt for brand-name medicines, but 50.4% of the population did not have an opinion in this respect. The strongest preference for GM was in the age group 45–55 (26.0%), whereas the strongest preference for brand-name medicines persisted in the age group > 55 (40.5%). Those who would prefer brand-name medicines were more often found in the group of other nationalities (Russians, Belarussians, Ukrainians, Poles, Lithuanians, etc.) (24.8%), compared to Latvians (18.3%).

Those who would prefer GM were more among respondents who have had positive/more positive than negative previous experience with GM (32.2%) (Table 3). Sociodemographic factors (sex, age, education and nationality) were not statistically significant to differentiate the respondents according to their previous experience with GM. The more positively respondents assessed their experience with GM, the more willing they were to choose GM instead of brand-name medicines (Spearman’s rho = 0.4, *p* < 0.001).

**Table 3:** Associations between experience and preferences, *n*=441^c^

	Would prefer GM,	No difference/do not have opinion,	Would prefer brand-name medicines
Negative/more negative than positive experience with GM	**0 (0.0%)**	**2 (8.0%)**	**23 (92.0%)**
Neutral experience	**32 (18.7%)**	87 (50.9%)	52 (30.4%)
Positive/more positive than negative experience with GM	**79 (32.2%)**	122 (49.8%)	**44 (18%)**

^c^Bolded text refers to responses that differ significantly from the total (adjusted standardized residuals value > 1.96 or < −1.96)

The more positively respondents assessed their experience with GM, the more willing they were to choose GM instead of brand-name medicines (Spearman’s rho = 0.4, *p* < 0.001) (see Table 4).

**Table 4:** Positive experience and willingness to choose GM

	Positive experience with GM
Willingness to choose GM, (Spearman’s rho)	0.4
Sig. (2-tailed)	0.0001

### Factors of importance when choosing between generic and brand-name medicines

**Fig. 1:**
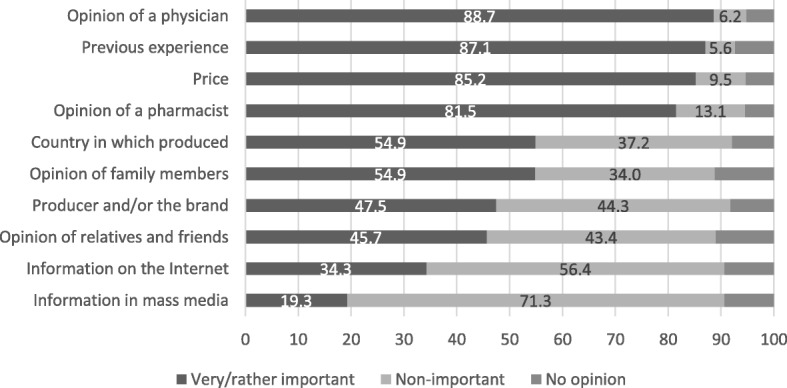
Factors of importance when choosing between GM and brand-name medicines, %, *n* = 1005

As seen in Fig. 1, four factors of the highest importance when choosing between GM and brand-name medicines were: [1] opinion of a physician – 88.7% of the respondents considered it as very or rather important; [2] previous experience with GM (87.1%), [3] price (85.2%), [4] opinion of a pharmacist – 81.5%.

### Information about generic medicines from different information sources

As shown in the Table 5, pharmacists provided more positive information about GM (47.2%), if compared to other sources of information in Latvia. Physicians of different specialties provided more negative information (7.7%), compared to the other sources.

**Table 5:** Information about generic medicines given by information sources (%), *n*=727^d^

Source	Positive/more positive than negative	Only neutral	Negative/more negative than positive	No information received/hard to say
Pharmacist	47.2	23.1	4.7	25.0
General practitioner	34.1	19.4	3.9	42.6
Physicians	28.0	18.2	7.7	46.1
Internet	26.4	15.1	5.4	53.1
TV, radio, mass media	18.6	16.4	6.4	58.6

^d^Respondents who were not informed about the existence of GM were excluded from the analysis

Small and medium correlation was found between information about GM from several sources and willingness to choose GM (see Table 6). The more positive information about GM was received from the following information sources, the more likely respondents were to choose GM instead of brand-name medicines - general practitioners (Spearman’s rho = 0.16), physicians from different specialties (Spearman’s rho = 0.18), pharmacists (Spearman’s rho = 0.15), family members (Spearman’s rho = 0.34), relatives and friends (Spearman’s rho = 0.35), as well as the Internet (Spearman’s rho = 0.18), *p* < 0.001.

**Table 6:** Positive information from different sources and willingness to choose GM

	GPs	Physician specialists	Pharmacists	Family members	Friends, relatives	TV, radio, newspapers	The Internet
Willingness to choose GM, (Spearman’s rho)	0.21	0.18	0.22	0.33	0.40	0.08	0.19
Sig. (2-tailed)	0.011	0.031	0.009	0.000	0.000	0.363	0.025

## Discussion

This is the first study in Latvia that examines awareness, experience and opinions of the population about GM. The results of the study showed that the majority of the population is informed about the availability of GM as an alternative to brand-name medicines. Women, higher educated and older patient groups were more informed compared to other groups. This might be explained by the fact that women and higher-educated people tend to have higher health literacy [22–25], and older people in Latvia are more exposed to the medicines’ reimbursement system in general, which distinguishes between generic and brand-name medicines, and to the NHSRL public campaigns promoting GM. The fact that men, people with primary and secondary education and nationalities other than Latvian are less informed may suggest that public information is not reaching these groups.

Approximately 44% of respondents in our study recalled previous experience with using GM. The actual number might be higher, as users might not always be aware whether they are using generic or brand-name medicines. The results show that the majority of those who recalled using GM rated their experience as positive or neutral. Only 5.7% of those surveyed reported their experience of using generics as negative. This finding is consistent with results reported by Drozdowska and Hermanowski in the Polish population where the majority were satisfied with using GM [21]. Negative experiences with GM safety or efficacy have been reported in other studies [15, 16]. This experience is not consistent with clinical studies; systematic reviews of equivalence between generics and brand-name medicines does not confirm differences in efficacy or safety [26, 27]. It is possible that patients’ negative experience of lower efficacy or side effects with GM might be influenced by nocebo effects [28].

Despite the high awareness and positive or neutral experience of those who had used GM, only about one fifth of the total number of respondents would prefer using GM, if they had to make a choice between GM and brand-name medicines in the future. This is different from the situation in Poland, where more than a half of the respondents were willing to opt for GM [25]. A systematic review by Colgan et al. also highlighted that a significant proportion of consumers held negative attitude towards GM which is a barrier for uptake of GM [16]. The negative attitude may be related to the fact that consumers do not consider GM as equal alternatives to brand-name medicines [29] and hold different myths about them [15]. Consistently with studies from Finland and Poland [17, 21], we did not find any associations between gender and preferences towards GM.

In the Latvian context, the strongest preference for brand-name medicines was in the age group over 55 years. This finding is unfortunate as most common chronic conditions are prevalent in this age group and representatives of the group might be beneficiaries of the pharmaceutical reimbursement system which promotes use of GM. These findings of preference for brand-name medicines and less willingness to use GM among older people are consistent with findings in other European countries like Finland, Switzerland and Belgium [17, 30, 31]. It would be useful to further explore reasons and identify best solutions to address mistrust towards GM in this age group.

Similar to other researchers [21, 15], we also found that the more positive GM users rated their experience, the more willing they were to choose GM if presented with a choice.

In our study, physician specialists were found to provide more negative information about GM compared to other information sources. It would be useful to explore reasons for this in further research. Possible explanations might be lack of knowledge about the concept of bioequivalence or specialists’ close relationships with the pharmaceutical industry. Studies have suggested that the payments from the pharmaceutical industry are associated with preferences for brand-name medicines [32, 33]. The systematic review by Dunne and Dunne suggests that physicians play a particularly important role in ensuring consumers’ confidence in GM [15]. The study that examined the relationship between patient beliefs and communication about GM with their providers, also concluded that the willingness to use GM is associated with positive communication with providers [34].

This study has the following limitations – as the survey was distributed as Computer Assisted Web Interviews, it did not reach the part of the population not using the Internet. It is estimated that approximately 21% of the Latvian population do not use Internet on a regular basis. The proportion of Internet nonusers differ significantly among the age groups. Within the age group 18–44 about 4% do not use Internet regularly, while within the age group 45–64 the share constitutes 28%, and within the age group 65–74 those are 64.5% [35]. To adjust to the criteria of a representative sample, data were weighted according to four parameters (gender, age, nationality and place of residence), but were not weighted according to education, therefore, the sample included a higher proportion of population with higher education than the general population in Latvia. It might have had impact on the study results. For instance, there are studies suggesting that population with higher education have more positive attitude towards GM and are more likely to choose generic substitution [15, 36].

## Conclusion

Although the majority of people in Latvia are informed about the existence of GM, and most of the users of GM rate their experience as positive, only about one fifth of the population would opt for GM. Certain groups like men, people with nationalities other than Latvian, people with primary and secondary education and elderly people were less aware or willing to use GM compared to other society members. This is a worrisome fact showing that there is a need to reconsider information that consumers receive from different information sources about GM. Choice of GM instead of usually more expensive brand-name medicines is particularly important in Latvia where a large part of the population lack access to medicines. As people in Latvia trust physicians and pharmacists in their choice between medicines, it is important that they provide objective and unbiased information about GM, as well as promote their use.
